# Identification of competing endogenous RNAs of the tumor suppressor gene PTEN: A probabilistic approach

**DOI:** 10.1038/s41598-017-08209-1

**Published:** 2017-08-10

**Authors:** Kourosh Zarringhalam, Yvonne Tay, Prajna Kulkarni, Assaf C. Bester, Pier Paolo Pandolfi, Rahul V. Kulkarni

**Affiliations:** 1 Department of Mathematics, University of Massachusetts Boston, Boston, MA 02125 USA; 2 Cancer Research Institute, Beth Israel Deaconess Cancer Center, Department of Medicine and Pathology, Beth Israel Deaconess Medical Center, Harvard Medical School, Boston, MA 02215 USA; 3 Cancer Science Institute of Singapore and Department of Biochemistry, Yong Loo Lin School of Medicine, National University of Singapore, Singapore, 117597 Singapore; 4 Department of Physics, University of Massachusetts Boston, Boston, MA 02125 USA

## Abstract

Regulation by microRNAs (miRNAs) and modulation of miRNA activity are critical components of diverse cellular processes. Recent research has shown that miRNA-based regulation of the tumor suppressor gene PTEN can be modulated by the expression of other miRNA targets acting as competing endogenous RNAs (ceRNAs). However, the key sequence-based features enabling a transcript to act as an effective ceRNA are not well understood and a quantitative model associating statistical significance to such features is currently lacking. To identify and assess features characterizing target recognition by PTEN-regulating miRNAs, we analyze multiple datasets from PAR-CLIP experiments in conjunction with RNA-Seq data. We consider a set of miRNAs known to regulate PTEN and identify high-confidence binding sites for these miRNAs on the 3′ UTR of protein coding genes. Based on the number and spatial distribution of these binding sites, we calculate a set of probabilistic features that are used to make predictions for novel ceRNAs of PTEN. Using a series of experiments in human prostate cancer cell lines, we validate the highest ranking prediction (TNRC6B) as a ceRNA of PTEN. The approach developed can be applied to map ceRNA networks of critical cellular regulators and to develop novel insights into crosstalk between different pathways involved in cancer.

## Introduction

MicroRNAs (miRNAs) are known to be critical components of tumor suppressive pathways and dysregulation of miRNAs is commonly observed in human cancers^1, 2^. Thus, genetic mechanisms that regulate the activity of miRNAs are expected to play important roles in cancer initiation and progression. Recent research has uncovered a novel mechanism for regulation of miRNA activity with direct relevance to cancer^3–6^. It has been postulated that RNA targets are not merely passive substrates for regulation by miRNAs; by virtue of their binding to miRNAs, they can serve as key regulators of cellular abundance of free miRNAs. Cellular RNAs can compete for binding to a shared set of miRNAs and thereby modulate miRNA-based regulation by acting as competing endogenous RNA (ceRNA) targets.

The proposed mechanism of ceRNA-based regulation has been experimentally validated in cases such as regulation of the tumor suppressor gene PTEN^4–10^. PTEN is one of the most commonly altered tumor suppressor genes in human cancers and inactivation of PTEN occurs in a wide range of tumors^11–13^. The observation that variations in PTEN levels have highly significant effects on cancer susceptibility^14^ underscores the importance of discovering and analyzing ceRNA-based mechanisms of controlling cellular PTEN levels. Following the original discovery of ceRNA-based regulation of PTEN, several groups have developed methods for genome-wide prediction of PTEN ceRNAs^5, 6, 9, 15, 16^. These approaches have focused on identifying ceRNAs based on: a) sequence-based features derived from the locations and binding affinities of different miRNA binding sites in 3′ UTR regions and b) analysis of co-expression data across multiple samples and tissues. While these approaches have resulted in the discovery of multiple PTEN ceRNAs, it is anticipated that the ceRNA network is more extensive and several potential PTEN ceRNAs are as yet undiscovered.

Although several recent studies demonstrate the effectiveness of regulation by ceRNAs^8, 17–19^, the ceRNA hypothesis has also generated controversy with regards to its physiological relevance^20, 21^. The controversy stems from experimental observations and computational models^22–24^ which indicate that remarkably high copy numbers of additional miRNA-binding sites are required to increase the expression of mRNAs repressed by miRNAs. As no single mRNA is expected to reach such high levels *in vivo*, it was argued that examples of ceRNAs under physiological conditions are likely to be rare. On the other hand, a recent study by ref. 25 demonstrates the ceRNA effect due to high levels of expression of the neuroblastoma master oncogene MYCN, which impacts regulation by the highly abundant let-7 miRNA family in MYCN-amplified neuroblastoma cells. This discovery also highlights the importance of identifying *potential* ceRNAs for a given target gene: transcripts which, provided they can be amplified to high levels (either naturally or by inducing them), can give rise to ceRNA-based regulation. In such cases, it is of interest to investigate sequence-based signatures determining the potential effectiveness of a transcript for ceRNA-based regulation.

One of the challenges in discovering potential ceRNAs is related to the problem of identification of miRNA binding sites. These sites are typically identified using target prediction algorithms, which are known to have high error rates^26^. Furthermore, even assuming that the binding sites have been accurately identified, it is not clear how to associate significance to features (derived from binding-site locations) that contribute to the efficacy of the RNA molecule to act as a ceRNA. To address these challenges, we have developed a bioinformatics approach that is based on a) identification of miRNA binding sites using PAR-CLIP^27^ and CLASH experiments^28^ and b) probabilistic approaches to associate statistical significance to features derived from the number and the spatial distribution of the binding sites. The restriction to high-affinity experimentally validated miRNA binding sites minimizes false positives in binding site identification. While this restriction suggests that some *bona fide* ceRNAs will be missed by our approach, it is expected that the method will lead to high-confidence predictions.

Our algorithm is general and can be applied to uncover the ceRNA network for any target gene. The application of this method to PTEN leads to several novel predictions, which indicate multiple potential links to other pathways involved in cancer. Interestingly, our highest-ranking prediction for a novel PTEN ceRNA is TNRC6B, which is known to play a role in post-transcriptional repression by miRNAs^4^. In a series of experiments in prostate cancer cell lines, we demonstrate that TNRC6B indeed functions as an effective ceRNA of PTEN. This experimental validation indicates an important link between miRNA-based regulation pathways and tumor suppressive pathways involving PTEN and suggests that ceRNA-based cross-regulation between different pathways can play important roles in cancer biology.

## Methods

Identification of ceRNAs of a given target gene can be thought of as a machine learning problem, where one would seek to identify patterns that can distinguish ceRNAs from other non-interacting RNAs. An essential characteristic of ceRNAs is their ability to efficiently compete for miRNA binding with the target gene^21, 29^. One of the key factors in the efficiency of miRNA titration is the number of miRNA regulators shared between the ceRNA and the target gene and the distribution of the corresponding binding sites, i.e. miRNA response elements (MREs)^30^. Correspondingly, our approach is based on identifying and analyzing sequence-based features derived from the locations of MREs in potential ceRNAs. Note that, besides sequence-based features, expression levels are also expected to play a key role in determining the ability of a transcript to act as a ceRNA. However, our focus is on identifying *potential* ceRNAs of PTEN (i.e. genes that can act as PTEN-ceRNAs when expressed at appropriate levels); correspondingly our approach focuses entirely on sequence-based features. We group miRNAs into miRNA families according to similarity in the seed region^21^; miRNAs that share the same seed region are considered as one family. Next, using PAR-CLIP experiments and miRNA expression profiles^31^, we identified the expressed miRNAs (miRNA families) in human prostate cell lines and calculated the location of their MREs on the 3′ UTRs of every protein coding gene expressed in human prostate cell lines. Expressed genes in human prostate cell lines were obtained by analyzing RNA-Seq data^32^. See section “Data Processing Pipeline” in Methods for details of the pipeline.

Having identified the locations and the number of MREs, the next step is analysis of the corresponding features that can be used to identify ceRNAs. Previous work has identified a set of sequence-based features derived from the locations of the MREs that can be used for prediction of ceRNAs^2, 5^. Trans-regulatory ceRNA crosstalk is expected to increase with increasing number of shared miRNAs between transcripts^5^. Correspondingly the number of MREs and the number of targeting families must be taken into consideration for identifying ceRNAs. However, as miRNAs have many targets and transcripts are typically targeted by multiple miRNA, it is expected that there will be a “background” overlap between transcript MREs. As such statistical significance of the overlap can be a better predictor of cross-talk than absolute shared numbers.

It should also be noted that the length of the transcript (or its 3′ UTR) plays a role in in the expected number of shared MREs. Previous studies^33, 34^ suggest that in scenarios where there are many predicted MREs in a candidate transcript, the MREs are spread over a relatively short spans and in clusters. Moreover, cooperativity in miRNA binding suggests that the relative spatial locations of MREs play a significant role in determining ceRNA efficacy^35–38^. Several studies have shown that there is an optimal range of spacing between MREs for effective regulation^35–38^, suggesting that the distance between the MREs and measures corresponding to spcaing of the MREs constitute important features.

**Figure 1:**
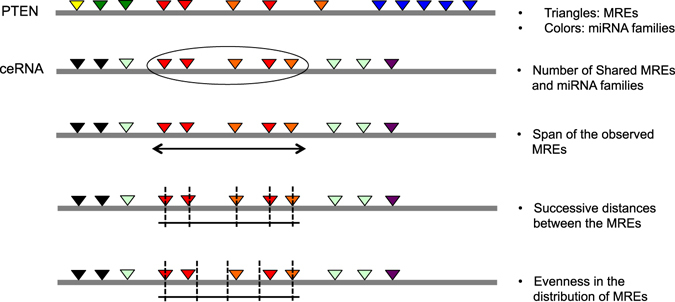
Features of ceRNAs. Figure shows a schematic representation of ceRNA features derived from MRE locations. Colors represent miRNA families and triangles represent MREs.

Motivated by these findings, we focus on the following features: 1) Statistical significance of the number of shared MREs between a candidate ceRNA and PTEN, 2) Statistical significance of the number miRNA families shared between the candidate ceRNA and PTEN, 3) Statistical significance of the span of the shared MREs, 4) Statistical significance of the distances between successive shared MREs and 5) Statistical significance of the degree of evenness in the distribution of the shared MREs. A novelty in the current work lies in the development of analytical probabilistic measures for quantification of statistical significance associated with features motivated by experimental studies. Figure 1 shows a schematic plot of the features. Additionally, we required that if a transcript acts as an effective ceRNA of PTEN, the converse should also be true, i.e., PTEN should act as an effective ceRNA of the transcript. For this reason, we inverted the role of PTEN and the transcripts and recalculated every feature. These features were multiplied by their corresponding features with flipped roles of PTEN and the putative ceRNA transcript.

### Features of ceRNAs

In what follows, *G* denotes the target gene of interest (e.g., PTEN) and *T* denotes a given candidate ceRNA transcript. We will refer to the set of miRNA families that target the gene *G* and their MREs as target-gene miRNA families and target-gene MREs respectively. Similarly, the set of miRNA families and their MREs that target *T* will be referred to as transcript miRNA-families and transcript MREs. The following two features assess the statistical significance of the observed number of shared MREs and miRNA families between the (*G*, *T*) pair.

#### Statistical significance of the observed MREs and miRNA families

The significance of the number of shared MREs between the gene *G* and the transcript *T* can be assessed using the binomial test. Each MRE on the transcript *T* that corresponds to a miRNA family shared by the target gene *G* can be viewed as a success in a sequence of Bernoulli trails corresponding to all observed MREs on the transcript *T*. The probability of success, *p*, is computed by the ratio of number of target gene miRNA families and the total number of miRNA families expressed in the cell line.

Let *K* denote the total number of considered target-gene miRNA families, *k* ≤ *K* the number of shared miRNA families between the target-gene and the transcript, *n* total number of transcript miRNA families and *N* total number of miRNA families overall. The significance of observing *k* number of shared families can be computed by a hyper-geometric test as follows:

$$Pr(X\ge k)=\sum _{i=k}^{K}\,\frac{(\begin{array}{c}K\\ i\end{array})\,(\begin{array}{c}N-K\\ n-i\end{array})}{(\begin{array}{c}N\\ n\end{array})}.$$

where *X* is a hyper-geometrically distributed random variable representing the number of shared miRNA families.

#### Statistical significance of the spatial position of MREs

In what follows capital letters denote random variables and the corresponding lower case letters denote an instance of the random variables. First we map the sequence length (i.e. 3′ UTR) and the target sites to the interval $[0,1]$. Let *n* denote the number of the observed target-gene MREs observed on a transcript *T*. The null hypothesis is that each target site is randomly drawn from a uniform distribution ${X}_{i}\sim {\rm{U}}\,\mathrm{(0},1)$. Support for this null hypothesis can be obtained by considering random shuffles of 3′ UTRs. For a shuffled 3′ UTR, the appearance of a MRE (as we go along the sequence) is expected to be a Poisson process (i.e. the probability of occurrence of a MRE is a constant along the sequence). Correspondingly, conditional on the presence of *n* MREs, we expect the statistics of their locations along the sequence will be indistinguishable from the order statistics of *n* points drawn from the corresponding uniform distribution. To test this, we generated 10,000 shuffles of the PTEN 3′ UTR and analyzed the distribution of one MRE (“GUGCAAA” from mir19 family) locations. Our analysis (data not shown) indicates the observed distribution is effectively a uniform distribution, thereby providing justification for the null hypothesis.

Drawing from the uniform distribution, we obtain a sequence of i.i.d. random variables ${\{{X}_{i}\}}_{i=1}^{n}$. Let ${X}_{\mathrm{(1)}}\le {X}_{\mathrm{(2)}}\cdots \le {X}_{(n)}$ be the order statistics of the sequence. In the following, we present the formulas for assessing the significance of the features. The details of the derivations are presented in the Supplementary Information.

#### Statistical significance of the observed span of MREs

Let $S={X}_{(n)}-{X}_{\mathrm{(1)}}$ be a random variable representing the span of target-gene MREs on the transcript and let *s*_0_ be the observed span of the sites. The p-value of the observed span *s*_0_ under the null hypothesis is given by the following formula (See Supplementary Information for details).

$$Pr(S\le {s}_{0})=Pr({X}_{(n)}-{X}_{\mathrm{(1)}}\le {s}_{0})=n\mathrm{(1}-{s}_{0}){s}_{0}^{n-1}+{s}_{0}^{n}$$

#### Statistical significance of the observed successive distances between the MREs

This feature is a measure of closeness of target-gene MREs on the transcripts *T*. Let *U*_*i*_ = *X*_(*i*+1)_ − *X*_(*i*)_, $i=1,\ldots ,n-1$ be a sequence of random variables representing the distances between successive sites and let ${d}_{1},\ldots ,{d}_{n-1}$ be the actual observed distances. It can be shown that (see Supplementary Information)

$$Pr({U}_{1}\le {d}_{1},\cdots {U}_{n-1}\le {d}_{n-1})=n!\prod _{j=1}^{n-1}\,{d}_{j}\,[1-\frac{1}{2}\sum _{i=1}^{n-1}\,{d}_{i}]$$

#### Statistical significance of evenness of the distribution of MREs

Let *X* = *X*_(*i*)_ and let *Y* = *X*_(*i*+1)_. It is straightforward to show that the density function of *U*_*i*_ = *X*_(*i*+1)_ − *X*_(*i*)_ is given by ${f}_{{U}_{i}}(t)=\tfrac{d}{dt}P({U}_{i}\le t)=n{\mathrm{(1}-t)}^{n-1}$. The mean of the above distribution, corresponding to the distance between successive MREs, is given by $E[{U}_{i}]=\tfrac{1}{n+1}$. We are interested in the deviation of the observed binding sites from the most evenly spaced distribution, i.e., when MREs are equally spaced at *i*/(*n* + 1), $i=1,\ldots ,n$. To measure this deviation, define ${Y}_{i}={({U}_{i}-\tfrac{1}{n+1})}^{2}$ and let $\bar{Y}=\tfrac{1}{n-1}{\sum }_{i=1}^{n-1}\,{Y}_{i}$. The expected value and the variance of *Y*_*i*_ can then be computed using the density function of *U*_*i*_ and are given by $E[{Y}_{i}]=\tfrac{n}{{(n+\mathrm{1)}}^{2}\,(n+\mathrm{2)}}$ and ${\rm{Var}}({Y}_{i})=\tfrac{4n\mathrm{(2}{n}^{3}+2{n}^{2}-3n+\mathrm{3)}}{{(n+\mathrm{1)}}^{4}\,{(n+\mathrm{2)}}^{2}\,(n+\mathrm{3)}\,(n+\mathrm{4)}}$. Note that these values are independent of *i*. We will denote these values by *E*[*Y*] and Var[*Y*] respectively. By the central limit theorem, we get that

$$\mathop{\mathrm{lim}}\limits_{n\to \infty }\frac{\bar{Y}-(n-\mathrm{1)}E[Y]}{{[(n-\mathrm{1)}{\rm{Var}}(Y)]}^{\mathrm{1/2}}}=\phi $$

where *φ* is the standard normal distribution from which we can approximate the p-value of the deviation from evenness.

#### Inverting the role of PTEN and the potential ceRNAs

We focus on transcripts for which the prediction that it can act as a ceRNA goes both ways, i.e. if a transcript acts as an effective ceRNA of PTEN, the converse must also be true, i.e., PTEN should act as an effective ceRNA of the transcript. For this reason, we flipped the role of PTEN and the transcripts and recalculated every feature. These features were multiplied by their corresponding features with PTEN as the main target. This constitutes our final set of 5 statistical features ${p}_{1},\ldots ,{p}_{5}$.

### Assessing relevance of features to miRNA-transctipt interactions

We analyzed the distribution of the special features calculated from MRE location obtained from bonafide miRNA-transcript interactions (as obtained from PAR-CLIP data) and compared the distributions to that of randomly generated MREs. Specifically, we simulated randomly distributed MREs on transcripts by selecting random locations on the MREs according to a uniform distribution. The number of MREs per transcript and the the transcript lengths were drawn according to the distribution of MRE numbers in bonafide interactions and actual lengths of the transcripts. Next, we assessed whether the distribution of features are significantly different between real and random MREs using the Kolmogorov Smirnov test. As expected, there is a significant difference between the distribution of the features (p-value < 2^−16^ for span and clustering and p-value < 2^−4^ for evenness). This result indicate that spacial location of MREs are indicative of miRNA-transcript interactions.

### Classification and ranking of PTEN ceRNAs

As no mRNAs have been validated as “non-ceRNAs” of PTEN and since the number of validated ceRNAs of PTEN is currently limited, standard supervised machine learning methods cannot be applied to predict new ceRNAs of PTEN. As such we devised a scoring function by computing the average value of −*log* of the main statistical features: $s=-\tfrac{1}{5}{\sum }_{i=1}^{5}\,\mathrm{log}\,{p}_{i}$. The empirical p-values of the predicted ceRNAs were then computed by examining the distribution of the scores.

### Extensions to other datasets and RNA classes

Although the current study is focused on PTEN and the MREs on its 3′ UTR using PAR-CLIP experiments, our code-base is general and can perform ceRNA predictions for any transcript from a user specified RNA class on 3′ UTR, 5′ UTR or the entire transcript. Moreover, we provide parsed PAR-CLIP and CLASH data that can be utilized in predictions. It should be noted that based on PAR-CLIP, PTEN contains 39 MREs in its 3′ UTR and 4 in the coding sequences, while PTEN contains only 2 MREs based on CLASH, both in its coding sequences. Consequently, our predictions for PTEN are limited to the 3′ UTR and the PAR-CLIP dataset. The code and the processed data are available to download at: http://markov.math.umb.edu:443/ceRNA/.

### Experimental Methods

#### Reagents

Reagents are as follows: anti-HSP90 antibody 61041 (Becton Dickinson); anti-PTEN antibody 9559 (Cell Signaling); siGENOME siRNA reagents for nontargeting 2 (siNC), PTEN (siPTEN), CNOT6L (siCNOT), TNRC6B (siTNRC) and Dharmafect 1 (Dharmacon); Trizol reagent, Dulbecco’s modified Eagle medium (DMEM), Opti-MEM reduced serum media, and fetal bovine serum (FBS) (Invitrogen); RNeasy mini kit (QIAGEN).

#### Cell Culture and Transfection

DU145 and PC3 cells were grown in DMEM plus 10% FBS, penicillin/streptomycin, and glutamine at 37 °C in a humidified atmosphere with 5% CO_2_. 22rv1 and BM1604 cells were grown in RPMI-1640 plus 10% FBS, penicillin/streptomycin, and glutamine at 37 °C in a humidified atmosphere with 5% CO_2_. For the transfection of siRNAs, cells were transfected with 100 nM siRNAs in 12-well dishes at a density of 100,000 cells per well. Transfection was performed with Dharmafect 1 (Dharmacon) according to the manufacturer’s recommendations.

#### RNA Extraction and Real-Time PCR

Total RNA was extracted from cells using Trizol reagent as per the manufacturer’s instructions and subsequently column purified with RNeasy kits (QIAGEN). cDNA synthesis was performed using the High Capacity cDNA Archive kit and real-time PCR was subsequently performed with Taqman probes according to the manufacturer’s instructions (Applied Biosystems).

#### Protein Extraction and Western Blot Analysis

Cells were washed in chilled PBS and lysed by incubating on ice for 10 min with RIPA lysis buffer containing protease inhibitors. Lysates were cleared by centrifugation at 4 °C for 15 min at full speed, and protein concentrations were determined using Bradford dye (Bio-Rad). 5 *μg* of total protein was size fractionated by SDS-PAGE on 4–12% Bis-Tris acrylamide NuPAGE gels in MOPS SDS running buffer (Invitrogen) and transferred to nitrocellulose membranes in NuPage transfer buffer (Invitrogen) containing 10% methanol. The membranes were then probed with specific primary antibodies.

#### Growth curve

At 14 hr post-transfection, DU145 and PC3 cells were trypsinized, resuspended, counted and seeded in separate 12-well plates at a final density of 15,000/well. Starting from the next day (d0), one plate per day was washed with PBS, fixed in 10% formalin solution for 10 min at room temperature, and then kept in PBS at 4 °C. After the final timepoint, wells were stained with crystal violet, washed with distilled water and lysed with 10% acetic acid. Optical density was read at 595 nm.

#### Statistical Analysis

Data were analyzed using unpaired Student’s t test. Values of *p* < 0.05 were considered statistically significant. *p < 0.05; **p < 0.01; ***p < 0.001. The mean ± SD of three or more independent experiments is reported.

### Data Processing Pipeline

**Figure 2:**
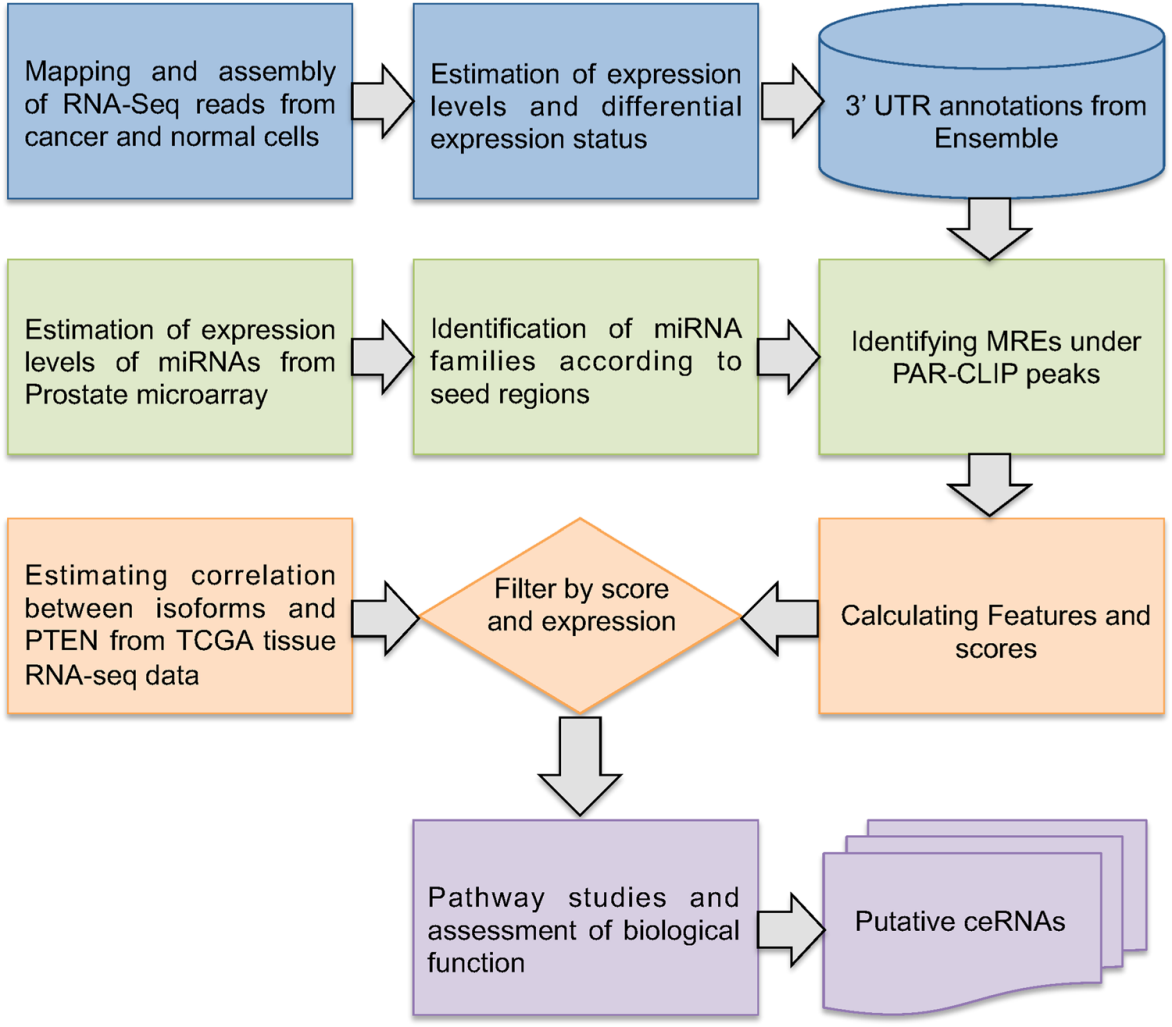
Data Processing pipeline. Figure shows a schematic representation of the data processing pipeline for prediction of putative ceRNAs.

We used previously validated miRNAs that are known to target PTEN based on MS2-RNA Immunoprecipitation (RIP)^5^ as well as miRNAs targeting PTEN as determined by PAR-CLIP experiments^16^ to identify and rank putative ceRNAs of PTEN as follows (See Fig. 2 for a schematic plot of the data processing and downstream classification pipeline).

#### Estimating gene expression levels

We obtain a total of 6 RNA-Seq datasets from DU145 prostate cancer cells^32^. The reads were mapped to the reference genome (hg38) and assembled using the HISAT2 pipeline^39^ and the relative abundance of the transcripts were estimated using featureCounts^40^ and the voom() function^41^ from the R Bioconductor package limma^42^. Genes with low expression levels (<10 fragment counts mapped to the region in all samples) were filtered out from the subsequent analysis. We used publicly available microarray data in prostate cells line to identify expressed miRNAs and estimate their expression levels^31^. miRNAs with expression level below the 5% quantile of the expression distribution of all miRNAs were considered as not expressed and removed from the subsequent analysis. A custom script was developed to categorize miRNAs into distinct families based on the similarity of the seed region.

#### Identification of miRNA binding sites

We obtained a total set of 15 PAR-CLIP datasets from AGO2 experiments^16^. The coordinates of the peaks of PAR-CLIP reads were mapped to hg38 using UCSC LiftOver tool. Ensemble annotations were used to identify the genomic coordinates of the targets sites on the 3′ UTRs of all the isoforms for every protein coding gene. A custom Python program was developed to scan the genomic locations under the PAR-CLIP peaks and match against the reverse complement of the seed region of the miRNA families. The families are defined according to the similarity of the seed regions and hence each match will uniquely determine a miRNA family. In our calculation we only considered high affinity sites (7mers, 8mers or 7mers + A matches). PTEN regulating miRNA families were identified through literature search and each overall miRNA family was given a status “Yes” or “No” according to whether the miRNA family targets PTEN. A detailed description of the computational pipeline is distributed with the code. CLASH data was obtained from the study of Helwak *et al*.^28^ and processed to map the MRE location to transcript-based relative locations.

#### Feature calculations and scoring of ceRNAs

Custom scripts were developed to calculate the features (see section “Features of ceRNAs”) from the genomic locations of MREs for every 3′ UTR. Furthermore, the features were recalculated by inverting the roles of PTEN and the transcript. These features were multiplied with their corresponding features with PTEN as the main target. The scoring function as described in Methods was calculated and empirical p-values for each predicted ceRNA were computed. Transcripts with low expression levels were filtered out (<10 fragment counts in all samples). It is hypothesized that optimal ceRNA-mediated cross-talk occurs at near equimolar equilibrium^29^. Correspondingly, we only considered transcripts with expression close to PTEN (<10 fold difference).

#### Enrichment analysis

GO term enrichment analysis was performed on the top ranking predicted ceRNAs. We performed GO term enrichment analysis of the top 100, 200, 300 and 400 predicted ceRNAs and performed GO terms enrichment analysis in each case. In our calculation we used the R topGO package. Reactome (http://www.reactome.org/) as well as as STRING-DB (http://string-db.org/) web-interfaces were used to perform similar enrichment studies on the top predictions.

## Results

### Comparison with other computational approaches for PTEN ceRNA prediction

In recent work, multiple databases and computational approaches have been developed for ceRNA identification and prediction^43–48^. It is of interest to note how our approach compares with some of these recent approaches for the specific case of PTEN ceRNA prediction. As detailed in the previous section, our method relies on PAR-CLIP data that most accurately, albeit stringently, identifies the MREs. Correspondingly, we expect that our approach using PAR-CLIP only identifies a subset of PTEN ceRNAs which have multiple high-confidence MRE sites that are identified by PAR-CLIP. At the significance level of 0.05, 6 of the 15 previously validated ceRNAs (as determined by the database miRSponge^46^) are predicted as significant according to our method. The total number of ceRNA predictions at this significance level is 244. The PAR-CLIP method and 90% are predicted as significant according to the Miranda method. Furthermore, we have also analyzed the prediction results from some other computational approaches and assessed the performance of these methods in predicting previously validated PTEN ceRNAs according to their reported score, rank or criteria.

In the case of cefinder^43^, since the significance, *p*-value, or probability are not reported in these methods (they report a score and a ranked list), we sought to calculate an empirical *p*-value based on the reported score distributions. cefinder makes their top 500 prediction along with their scores available for download. We approximated the *p*-value of the score of each ceRNA by computing the frequency of the more extreme examples (examples with higher or equal scores) divided by the total number of examples. According to the empirical p-values, cefinder correctly predicts 4 of the 15 ceRNAs. The total number of predictions that pass the significance level 0.05 are 22.

In case of starBase^44^, 10 of the 15 previously validated ceRNAs are correctly predicted as ceRNAs. The total number of predictions at the significance level 0.05 is 1412. It should be noted that starbase’s main measure of ceRNAs is essentially based on the number of shared miRNA families between PTEN and the gene. This is exactly our feature number 2. Since we have included 4 other biologically relevant features, it is expected that our method should yield more accurate results.

Finally, we examined the ceRNA prediction program Hermes^15^. Hermes predicts 185 PTEN ceRNAs. Of previously validated ones, 2 are correctly predicted by Hermes.

### Prediction of potential PTEN ceRNAs

We analyzed multiple datasets from PAR-CLIP experiments in conjunction with RNA-Seq data and derived a set of probabilistic features to identify characteristics of PTEN ceRNAs and to associate scores with putative ceRNAs (see Methods). We focused on the upper 98% quantile of the predicted ceRNAs which corresponds to the highest-scoring 170 genes. We took the top 170 predictions and performed enrichment studies to see if the top predictions are involved in specific cellular processes. For example a GO term enrichment analysis of the top 170 predictions showed that the top candidates were enriched in processes such as cellular metabolism and gene silencing by miRNAs. Note that the 98% quantile was chosen to focus on the top-ranking predictions. We also considered different values of the cutoff and the results obtained are reported in separate GO term tables available on the webpage. Briefly, we took the top 100, 200, 300, 400, 500 predicted ceRNAs and performed GO terms enrichment analysis in each case. The number of significant GO terms (at 0.01 level) in each case are 79, 191, 220, 192, 218, respectively. Among these 36 GO terms are in common which include terms such as “cell cycle process”, “cellular metabolism” and “gene silencing”. The full list of individual GO term enrichment analysis results as well as detailed prediction tables are available to download at http://markov.math.umb.edu:443/ceRNA/.

Further enrichment analysis on top predictions list using public databases such as STRING DB (http://string-db.org/)^49^ and Reactome (http://www.reactome.org/)^50^ revealed interesting potential connections to multiple pathways such as circadian clock and apoptosis, as highlighted in Table 1.

**Table 1:** A subset of PTEN ceRNA predictions organized in different categories based on Reactome analysis.

Category	Gene	Reactome pathway
1	TNRC6A, TNRC6B, TNRC6C, DICER1, AGO1	miRNA processing and regulation
2	RNF38, TP53INP1, HIPK2	associated with P53
3	CLOCK, RORA	Circadian clock
4	XIAP, CASP8, TAOK1, PDCD10, SOCS6, BCL2L11	Apoptosis regulators
5	ATXN1L, ATXN1	Notch signaling

Analysis of the interaction network for the predictions based on STRING DB (http://string-db.org/)^49^ indicated that there were significantly more interactions (528) than expected from a random selection of genes (196). Analysis of functional enrichments in biological processes categorized using GO terms by STRING revealed multiple connections to metabolic processes and regulation of metabolism; suggesting that the PTEN-ceRNA network could play a role metabolic reprogramming during cancer.

**Figure 3:**
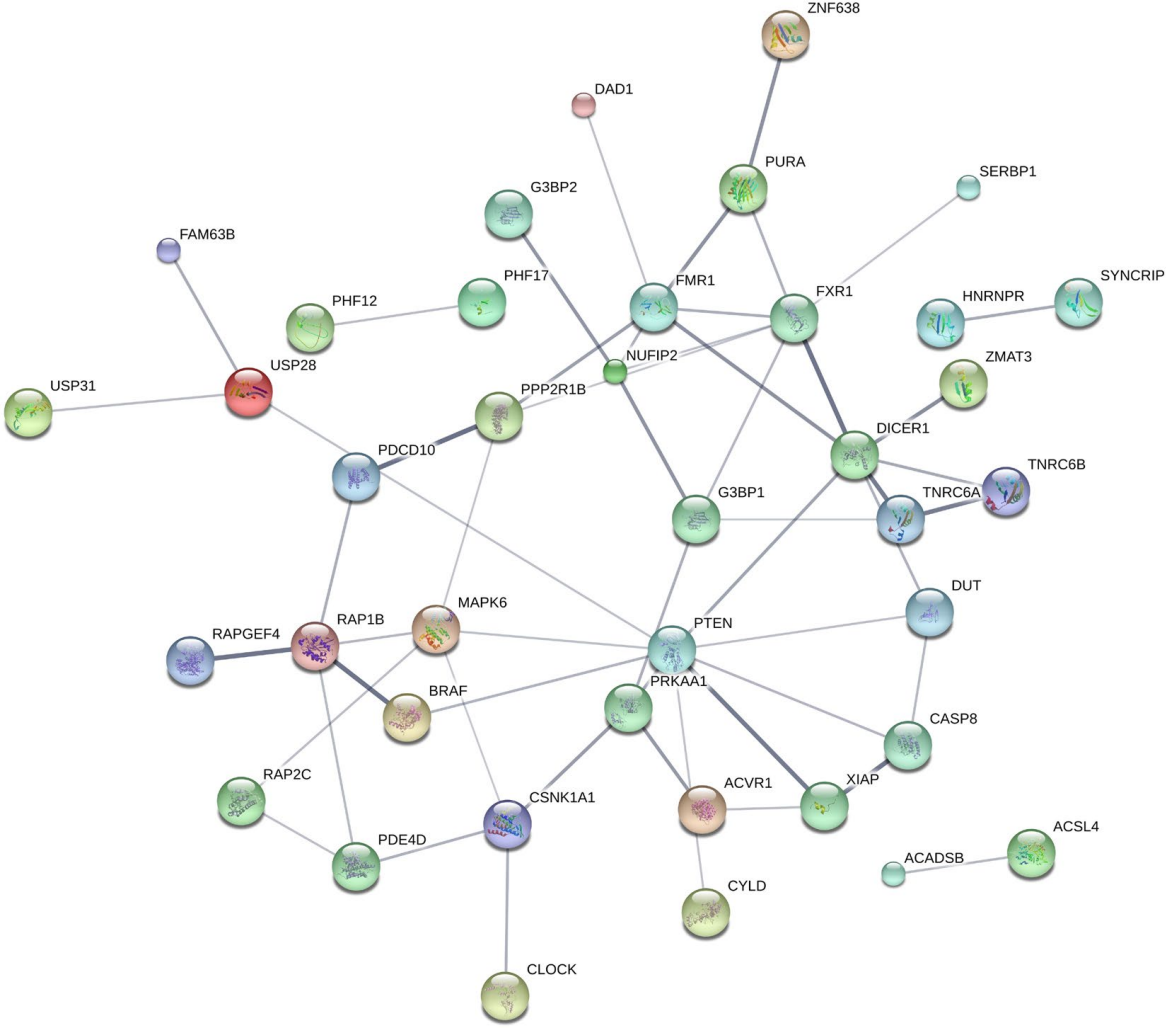
ceRNA network of PTEN. Figure shows the protein-protein interaction (PPI) of PTEN and its top 100 predicted ceRNAs, showing only the connected components of the network. All genes are among the top 100 predicted ceRNAs and genes with PPI connection to at least one other ceRNA or PTEN are depicted. The PPI network is obtained from the STRINGD DB database. The thickness and the darkness of the edge correspond to confidence in the PPI interaction.

Figure 3 represents the network of interaction between PTEN and the top 100 predicted ceRNAs as generated by STRING DB, showing only the connected nodes in the network. As indicated in the Figure, there are multiple ceRNA predictions for proteins known to interact with PTEN (e.g. BRAF and XIAP) as well as ceRNA predictions for important cellar regulators that were previously unconnected to PTEN (e.g. CLOCK).

**Figure 4:**
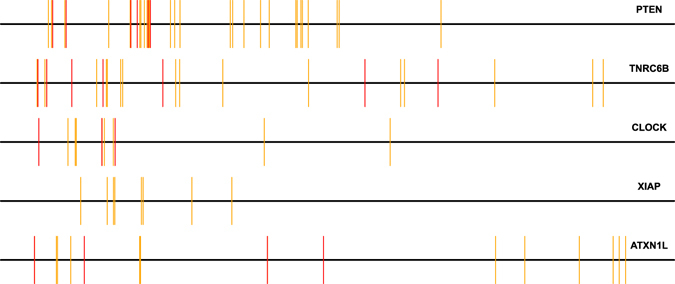
Distribution of MREs. Figure shows the location of MREs from PTEN targeting miRNAs on selected top candidates. The 3′ UTR sequence has been mapped to the the interval $[0,1]$ and the location of MREs are scaled accordingly. Red lines indicated MREs from experimentally validated PTEN miRNAs and orange bars are form PTEN targeting miRNAs as determined by PAR-CLIP experiments.

Figure 4 shows the distribution of MREs from miRNAs targeting PTEN on some of the top predictions. The 3′ UTR sequence has been mapped to the the interval $[0,1]$ and the location of MREs are scaled accordingly.

In order to validate our approach, we focused on experimentally testing the top scoring prediction, TNRC6B, as a ceRNA for PTEN. Notably, this prediction is consistent with previous results using a transposon-based mutagenesis screen^4^; however detailed experiments validating the ceRNA effect in human prostate cancer cell lines have not been done. We carried out experiments demonstrating reciprocal miRNA-mediated modulation of PTEN and TNRC6B levels and quantifying its impact on cell proliferation, as described below.

### Modulation of PTEN levels using TNRC6B as a ceRNA

**Figure 5:**
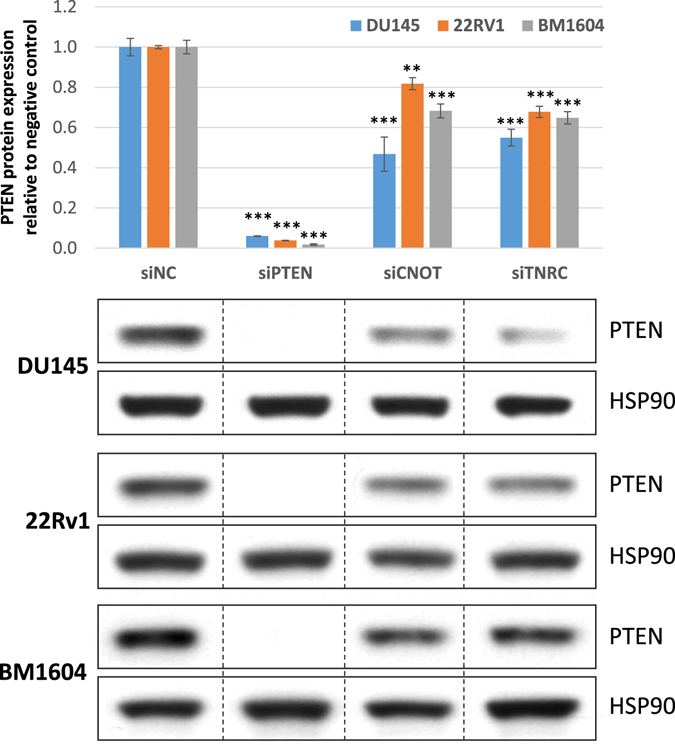
Predicted PTEN ceRNA TNRC6B modulates PTEN expression. Western blot for PTEN protein levels in prostate cancer cell lines transfected with siRNA against predicted ceRNA TNRC6B (siTNRC), validated ceRNA CNOT6L (siCNOT), PTEN (siPTEN) and a non-targeting control (siNC). Quantitation of the western analyses is shown above, and representative blots below.

As our data processing pipeline utilized data from prostate cancer, we first investigated the ability of this predicted PTEN ceRNA TNRC6B to modulate endogenous PTEN levels in several commonly used prostate cancer cell lines which express wild-type PTEN, DU145, 22rv1 and BM1604 (See Fig. 5). A significant reduction in PTEN protein levels was observed in DU145, 22rv1 and BM1604 cell lines after siRNA knockdown of the TNRC6B transcript, similar to that seen after depletion of CNOT6L, a validated PTEN ceRNA^4, 5^.

### Modulation of TNRC6B levels using PTEN as a ceRNA

**Figure 6:**
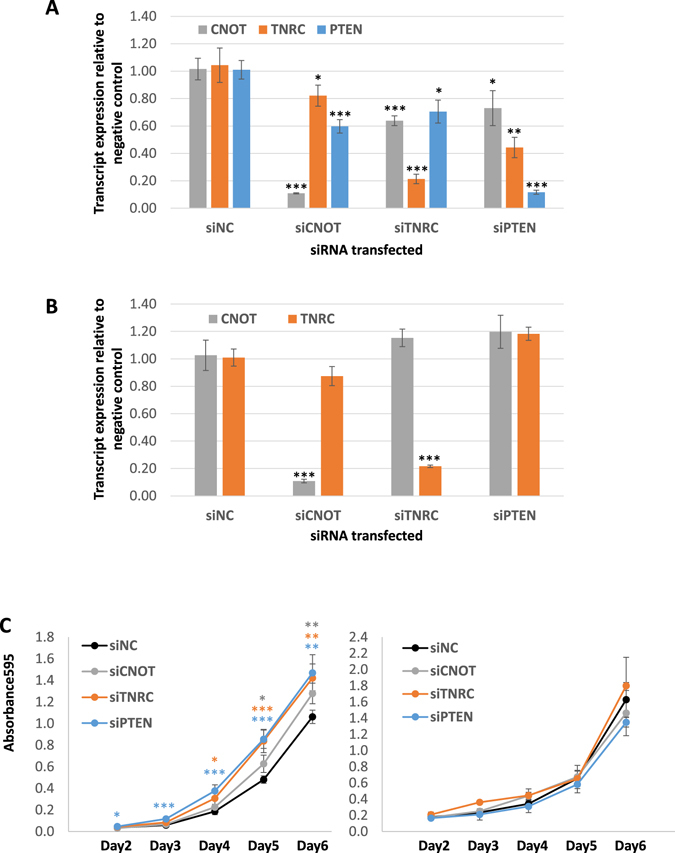
Crosstalk between PTEN and PTEN ceRNAs is reciprocal. (**A**,**B**) Real-time PCR analyses of changes in transcript expression of PTEN and PTEN ceRNAs after transfection with siRNA against TNRC6B, CNOT6L and PTEN relative to the non-targeting control in DU145 (**A**) and PC3 (**B**) cells. (**C**) Proliferation curve of DU145 (left) and PC3 (right) transfected with siRNAs against TNRC6B and PTEN. siPTEN and siTNRC6B result in a significant increase in growth relative to the siNC control in DU145 but not in PC3 cells.

We have hypothesized that ceRNA interactions are mutually reciprocal. To investigate this in the context of the PTEN, TNRC6B and CNOT6L network, we examined the effect of downregulation of PTEN transcript on TNRC6B and CNOT6L transcript expression and vice versa. We could not perform this experiment at the protein level due to the lack of specific antibodies for both CNOT6L and TNRC6B^5^. However, we observed that siRNA knockdown of PTEN significantly reduced expression of CNOT6L and TNRC6B transcripts in DU145 cells (Fig. 6A). Similarly, TNRC6B downregulation reduced expression of PTEN and CNOT6L transcripts, and CNOT6L downregulation reduced expression of PTEN and TNRC6B transcripts. To ascertain whether the crosstalk between TNRC6B and CNOT6L was PTEN-dependent, we repeated the experiment in the prostate cancer cell line PC3, which is PTEN-null. In PC3 cells, knockdown of TNRC6B did not have a significant effect on CNOT6L transcript levels, and vice versa (Fig. 6B). Taken together, these data suggest that the co-regulation between TNRC6B and CNOT6L is at least partially PTEN-dependent, and that PTEN, TNRC6B and CNOT6L crosstalk in a mutually reciprocal manner. We note, however, that it is possible that the lack of crosstalk in PC3 cells, instead of being PTEN dependent, could be linked to the expression levels of the TNRC6B and CNOT6L transcripts.

### Effect of TNRC6B, acting as a PTEN ceRNA, on cell proliferation

We next determined whether TNRC6B possessed tumor suppressive properties by evaluating its effect on cell proliferation. Downregulation of TNRC6B in DU145 cells with wild-type PTEN resulted in a significant increase in cell proliferation similar to that observed with downregulation of PTEN (Fig. 6C, left panel). Downregulation of either PTEN or TNRC6B in PTEN-null PC3 cells did not have a significant effect on cell proliferation, suggesting that the effect of TNRC6B on cell proliferation is at least partly due to its PTEN ceRNA activity.

## Conclusions and Future Work

The discovery of ceRNA-based cross-regulation of PTEN has marked the emergence of a new regulatory layer in cellular control of gene expression. This indirect mechanism of gene regulation is expected to play important roles both in normal cellular functions as well as in dysregulation leading to disease. The development of quantitative models for identifying ceRNAs, as proposed in this work, will significantly impact current and future research aimed at understanding the role of this mode of regulation in diverse cellular processes. A major challenge in identifying potential ceRNAs is the problem of identifying miRNA binding sites and associating statistical significance to the features derived from the locations of the binding sites. In this work, we present approaches and derive results addressing these issues.

The novelty of our approach lies in the development of analytical probabilistic measures for quantification of statistical significance associated with features motivated by experimental studies. Furthermore, the approach developed can be used in combination with more general methods to determine and model miRNA/mRNA interactions. More specifically, it can serve as input in generating dynamic models of ceRNA networks that take into account cellular levels of miRNAs and RNA transcripts^15, 23, 51–54^.

While the focus of this work is on predicting ceRNAs of PTEN, the approach and results are general and can be applied to predict potential ceRNAs for other cellular genes of interest. In particular, the approach developed can be replicated to uncover ceRNA networks of key cellular genes involved in cancer and to analyze potential overlaps between these networks. Indeed, we provide a general purpose code for performing ceRNA predictions for any transcript. The code can perform the predictions based on user defined regions on the transcript (e.g., 3′ UTR, 5′ or the entire transcript) as well as user specified RNA classes (e.g., protein coding, lincRNA, etc). It should be noted that most of PTEN MREs are located on its 3′ UTR based on PAR-CLIP (39 MREs in the 3′ and 4 in coding sequences). The CLASH dataset only shows 2 PTEN MREs located in coding sequences. However, this is not necessarily the case for other transcripts. MRE prediction software algorithms may also show additional MREs in other regions.

Our experimental validation of TNRC6B as a PTEN ceRNA, in combination with predictions for other pathway components (Table 1), points towards an intriguing link between pathways of miRNA processing/regulation and PTEN. The ceRNA effect indicates that, under appropriate cellular conditions, reduction of PTEN mRNA levels can result in lowering of TNRC6B levels, as demonstrated in this work. On the other hand, reduction of TNRC6B is also expected to lower the activity of miRNAs, presumably leading to higher levels of PTEN. Since this is not seen in the experiments, our observations suggest that the ceRNA effect dominates over any effect that reduction of TNRC6B levels may have on miRNA activity. Furthermore, reductions in other components of the miRNA processing/regulation pathway, as predicted by our results (Table 1), would be expected to lead to a global reduction in cellular miRNA activity. Given that several experiments have shown that global suppression of miRNA expression is oncogenic^55^, it would be of considerable interest to explore the cellular effects of such ceRNA-based reduction of miRNA activity by PTEN.

The current predictions for PTEN ceRNAs also include several potential linkages to critical cellular pathways (e.g. circadian clock genes and TP53 regulated genes) and it would be of interest to explore these connections in future work. Large-scale validation of the predicted targets can establish linkages to other cancer-associated pathways and lead to novel insights into the role of ceRNA-based regulation in tumorigenesis. Such approaches can also be used to combine sequence-based features (the focus of the current work) with information theoretic and co-expression-based features (e.g., ref. 15) to develop an integrated approach for the identification of ceRNA networks.

## Electronic supplementary material

Supplementary Information
