# Supplementary material for: Identification of competing endogenous RNAs of the tumor suppressor gene PTEN: A probabilistic approach
Source: Sci Rep. 2017 Aug 10;7:7755. doi: 10.1038/s41598-017-08209-1 (PMC5552881; doi:10.1038/s41598-017-08209-1)
Supplement: Supplementary file 1 — Supplementary Information [file 41598_2017_8209_MOESM1_ESM.pdf]

Supplementary Information: Identification of competing endogenous RNAs of the tumor suppressor gene PTEN: A probabilistic approach

Kourosh Zarringhalam[1,*], Yvonne Tay[2,3], Prajna Kulkarni[4], Assaf C. Bester[2], Pier Paolo Pandolfi[2], and Rahul V. Kulkarni[4]

[1]Department of Mathematics, University of Massachusetts Boston, Boston MA 02125, USA.
[2]Cancer Research Institute, Beth Israel Deaconess Cancer Center, Department of Medicine and Pathology, Beth Israel Deaconess Medical Center, Harvard Medical School, Boston, MA 02215, USA.
[3]Cancer Science Institute of Singapore and Department of Biochemistry, Yong Loo Lin School of Medicine, National University of Singapore, Singapore 117597.
[4]Department of Physics, University of Massachusetts Boston, Boston MA 02125, USA.
[*]kourosh.zarringhalam@umb.edu

ABSTRACT

Regulation by microRNAs (miRNAs) and modulation of miRNA activity are critical components of diverse cellular processes. Recent research has shown that miRNA-based regulation of the tumor suppressor gene PTEN can be modulated by the expression of other miRNA targets acting as competing endogenous RNAs (ceRNAs). However, the key sequence-based features enabling a transcript to act as an effective ceRNA are not well understood and a quantitative model associating statistical significance to such features is currently lacking. To identify and assess features characterizing target recognition by PTEN-regulating miRNAs, we analyze multiple datasets from PAR-CLIP experiments in conjunction with RNA-Seq data. We consider a set of miRNAs known to regulate PTEN and identify high-confidence binding sites for these miRNAs on the 3′ UTR of protein coding genes. Based on the number and spatial distribution of these binding sites, we calculate a set of probabilistic features that are used to make predictions for novel ceRNAs of PTEN. Using a series of experiments in human prostate cancer cell lines, we validate the highest ranking prediction (TNRC6B) as a ceRNA of PTEN. The approach developed can be applied to map ceRNA networks of critical cellular regulators and to develop novel insights into crosstalk between different pathways involved in cancer.

Detailed derivation of mathematical results

In this section we present the details of the mathematical models presented in the main text.

Statistical significance of the spatial position of MREs: Drawing from the uniform distribution, we obtain a sequence of i.i.d random variables $\{X_i\}_{i=1}^{n}$. Let $X_{(1)} \leq X_{(2)} \cdots \leq X_{(n)}$ be the order statistics of the sequence. In the following, we present the formulas for assessing the significance of the features. We start by reviewing some results on the distribution of order statistics that are needed to assess significance of the features discussed[1]. The joint distribution of the ordered statistics is given by

$$f_{(X_{(1)},\cdots,X_{(n)})}(x_1,\cdots,x_n) = n!$$

where $f(x)$ is the density function of the uniform distribution over the interval $[0,1]$. The density functions for the order statistics $X_{(j)}$ and the joint random variable $(X_{(i)},X_{(j)})$, $j>i$, are given by

$$f_{(X_{(i)},X_{(j)})}(x_i,x_j) = \binom{n}{i-1,\, j-i-1,\, n-j} F(x_i)^{i-1} \times (F(x_j)-F(x_i))^{j-i-1}(1-F(x_j))^{n-j} f(x_i) f(x_j)$$

where $F(x)$ denotes the cumulative distribution of the uniform distribution over the interval $[0,1]$.

Statistical significance of the observed span of MREs: Let $S = X_{(n)} - X_{(1)}$ be a random variable representing the span of target-gene MREs on the transcript and let s_0 be the observed span of the sites. The p-value of the observed span s_0 under the null hypothesis can be calculated as follows.

$$Pr(S \le s_0) = Pr(X_{(n)} - X_{(1)} \le s_0) = \iint_{x_n - x_1 \le s_0} f_{(X_{(n)},X_{(1)})}(x_1,x_n)dx_1dx_n$$
$$= \int_0^1 \int_{x_1}^{x_1+s_0} \frac{n!}{(n-2)!}(F(x_n)-F(x_1))^{n-2} \times f(x_1)f(x_n)dx_ndx_1 = n(1-s_0)s_0^{n-1} + s_0^n$$

Statistical significance of the observed successive distances between the MREs: This feature is a measure of closeness of target-gene MREs on the transcripts T. Let $U_i = X_{(i+1)} - X_{(i)}$, $i = 1,\cdots,n-1$ be a sequence of random variables representing the distances between successive sites and let $d_1,\cdots,d_{n-1}$ be the actual observed distances. We would like to compute the probability

$$Pr(U_1 \le d_1,\cdots,U_{n-1} \le d_{n-1}) = \int \cdots \int_D f_{(X_{(1)},\cdots,X_{(n)})}(x_1,\cdots,x_n)dx_1\cdots dx_n$$

where D is the region specified by $0 \le x_1 \le 1, 0 \le x_2 - x_1 \le d_1,\cdots,0 \le x_n - x_{n-1} \le d_{n-1}$. Using change of variables $u_i = x_{i+1} - x_i$, the integration region can be specified by $u_1 \le d_1,\cdots,u_{n-1} \le d_{n-1}$ and $x_1 \le 1-u$ where $u = u_1 + \cdots + u_{n-1}$. We can then compute the probability as follows

$$Pr(U_1 \le d_1,\cdots U_{n-1} \le d_{n-1}) = n! \int_0^{d_1} \cdots \int_0^{d_{n-1}} \int_0^{1-u} dx_1du_{n-1}\cdots du_1$$
$$= n! \int_0^{d_1} \cdots \int_0^{d_{n-1}} (1-u_1-\cdots-u_{n-1})du_{n-1}\cdots du_1$$

Writing the integrand $(1-u_1-\cdots-u_{n-1})$ as $(1-u_1)+(1-u_2)+\cdots+(1-u_{n-1})-(n-2)$ and decomposing the above integral, we obtain the following formula

$$Pr(U_1 \le d_1,\cdots U_{n-1} \le d_{n-1}) = n! \prod_{j=1}^{n-1} d_j \left[1 - \frac{1}{2}\sum_{i=1}^{n-1} d_i\right]$$

Statistical significance of evenness of the distribution of MREs: Let $X = X_{(i)}$ and let $Y = X_{(i+1)}$. The joint density function of (X,Y) is given by

$$f_{(X,Y)}(x,y) = \frac{n!}{(i-1)!(n-i-1)!}x^{i-1}(1-y)^{n-i-1}$$

Let $U = Y - X$. Then

$$P(U \le d) = \frac{n!}{(i-1)!(n-i-1)!} \times \int_0^d \int_0^{1-u} x^{i-1}(1-u-x)^{n-i-1}dxdu$$

Using change of variables $x = \xi(1-u)$, we get

$$P(U \le d) = \frac{n!}{(i-1)!(n-i-1)!} \int_0^d (1-u)^{n-1} \times \int_0^1 \xi^{i-1}(1-\xi)^{n-i-1}d\xi du$$
$$= \frac{n!}{(i-1)!(n-i-1)!}\beta(i,n-i)\int_0^d (1-u)^{n-1} = 1-(1-d)^n$$

where $\beta(i,n-i) = \dfrac{(i-1)!(n-i-1)!}{(i+n-i-1)!}$ is the β function. Hence the density function of $U_i = X_{(i+1)} - X_{(i)}$ is given by

$$f_{U_i}(t) = \frac{d}{dt}P(U_i \le t) = n(1-t)^{n-1}$$

The mean of the above distribution, corresponding to the distance between successive MREs, is given by $E[U_i] = \frac{1}{n+1}$. We are interested in the deviation of the observed binding sites from the most evenly spaced distribution, i.e, when MREs are equally

spaced at $i/(n+1)$, $i = 1, \cdots, n$. To measure this deviation, define $Y_i = (U_i - \frac{1}{n+1})^2$ and let $\bar{Y} = \frac{1}{n-1} \sum_{i=1}^{n-1} Y_i$. The expected value and the variance of Y_i can then be computed using the density function of U_i and are given by

$$E[Y_i] = \frac{n}{(n+1)^2(n+2)}$$

and

$$\mathrm{Var}(Y_i) = \frac{4n(2n^3 + 2n^2 - 3n + 3)}{(n+1)^4(n+2)^2(n+3)(n+4)}$$

Note that the above values are independent of i. We will denote these values by $E[Y]$ and $\mathrm{Var}[Y]$ respectively. By the central limit theorem, we get that

$$\lim_{n\to\infty} \frac{\bar{Y} - (n-1)E[Y]}{[(n-1)\mathrm{Var}(Y)]^{1/2}} = \varphi$$

where φ is the standard normal distribution from which we can approximate the p-value of the deviation from evenness.

References

1. David, H. A. & Nagaraja, H. N. *Order statistics*, vol. 3 (Wiley Online Library, 1970).
